# Supplementary material for: HCMV-encoded US7 and US8 act as antagonists of innate immunity by distinctively targeting TLR-signaling pathways
Source: Nat Commun. 2019 Oct 11;10:4670. doi: 10.1038/s41467-019-12641-4 (PMC6789044; doi:10.1038/s41467-019-12641-4)
Supplement: Supplementary file 1 — Supplementary Information [file 41467_2019_12641_MOESM1_ESM.pdf]

Supplementary Information

HCMV-Encoded US7 and US8 Act as Antagonists of Innate Immunity by Distinctively Targeting TLR Signaling Pathways

Park et al.

Supplementary Figure 1. Park et al.

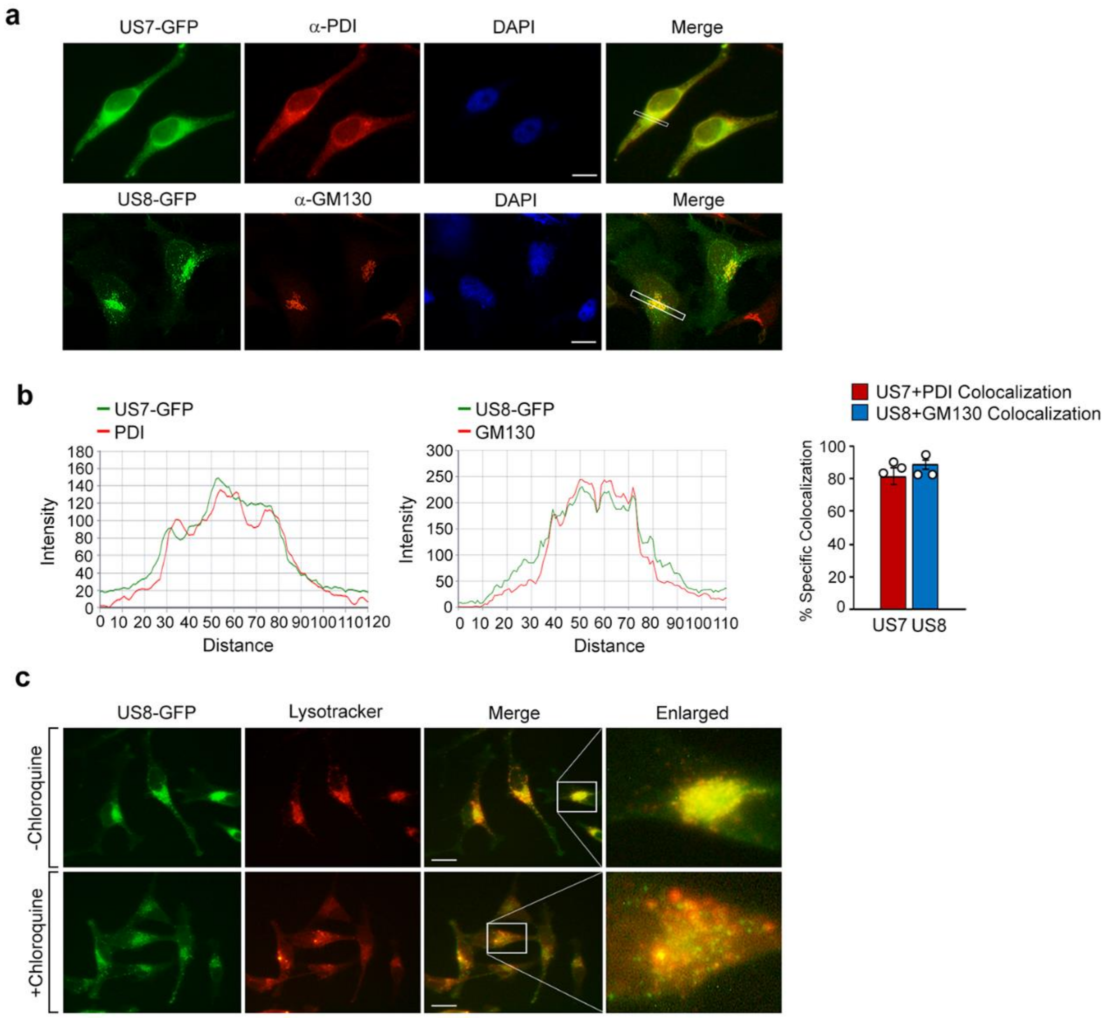

Supplementary Figure 1. Subcellular localization of HCMV US7 or US8 (**a**) HeLa cells expressed US7-GFP or US8-GFP were stained with an ER marker (anti-PDI) or Golgi marker (anti-GM130). The nuclei were stained with DAPI. Scale bars, 10 µm. (**b**) Quantification of US7 associated with TLR3 or TLR4 is included. The fluorescence intensity (FI) of the IFA images was quantified using the Zen software (Carl Zeiss; http://zeiss.com). Data are given as average of FI per cell in the selected fields. (**c**) HeLa cells were transfected with US8-GFP and stained with lysosomal marker (lysotracker) in the absence or presence of 100 µM chloroquine for 4 h. Data are representative of three independent experiments.

Supplementary Figure 2. Park et al.

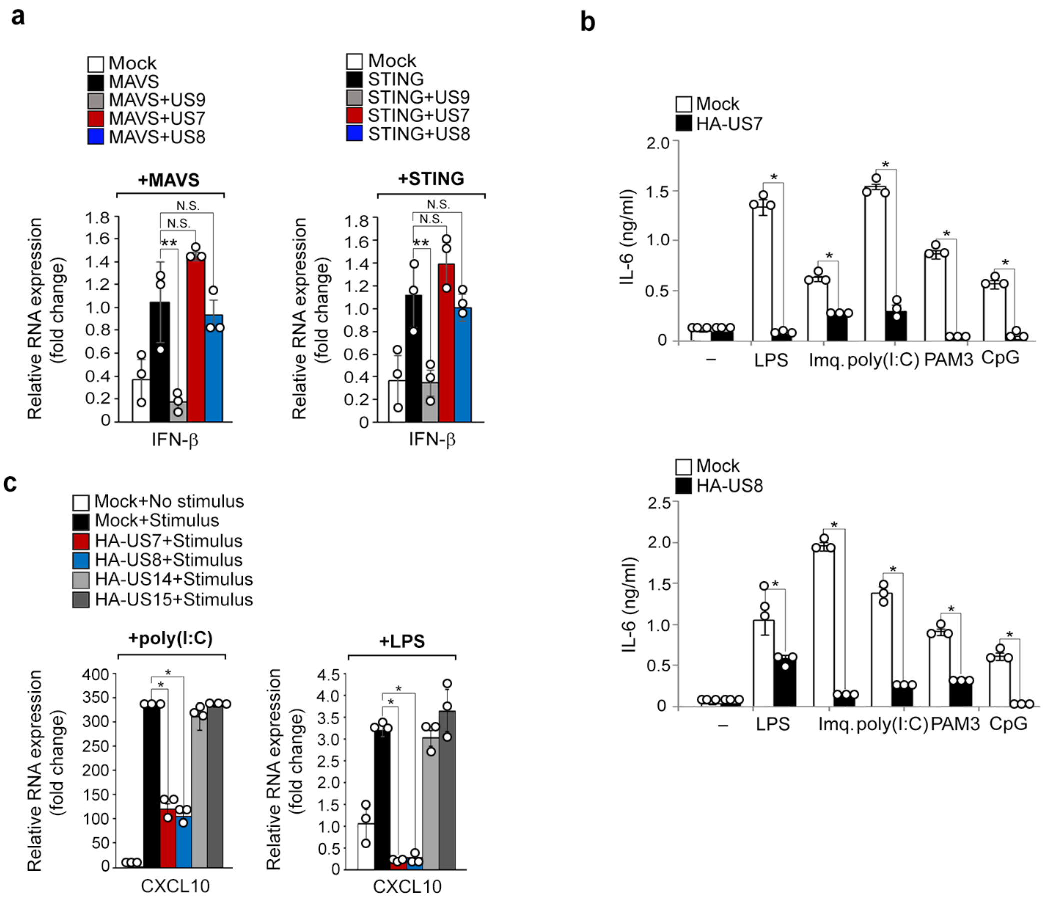

Supplementary Figure 2. US7 and US8 affect TLRs-mediated signaling, but not MAVS or STING pathway. (**a**) The effect of MAVS and STING-mediated IFN-β expression in US7- or US8-expressin cells. Empty vector-, US7-GFP-, US8-GFP- or US9-GFP-expressing HEK 293T cells were stimulated by MAVS and STING overexpression. IFN- β mRNA expression levels were measured by qPCR. ***P*<0.05 (Student's *t*-test). (**b**) US7 and US8 downregulate TLR-mediated IL-6 mRNA production. THP-1 cells expressing empty vector, US7, or US8 were stimulated by 100 ng ml^{-1} Pam3CSK4, 5 µg ml^{-1} LPS, 10 µg ml^{-1} poly(I:C), 5 µg ml^{-1} imiquimod, or 5µM CpG-DNA for 12 h. IL-6 secretion levels were measured by ELISA. **P*<0.001 (Student's *t*-test) (**c**) US7 and US8 suppress antiviral gene expression mediated by TLR3 and HeLa cells stably expressing TLR3-Myc and TLR4-Myc were transfected with empty vector, HA-US7, HA-US8, HA-US14, or HA-US15. Cells were then stimulated by 10 µg ml^{-1} poly(I:C) or 5 µg ml^{-1} LPS for 12 h. The indicated gene expression was measured by qPCR. **P*<0.001, ***P*<0.05 (Student's *t*-test). Data are representative of three independent experiments and are presented as means ± s.d. in **a-c**.

Supplementary Figure 3. Park et al.

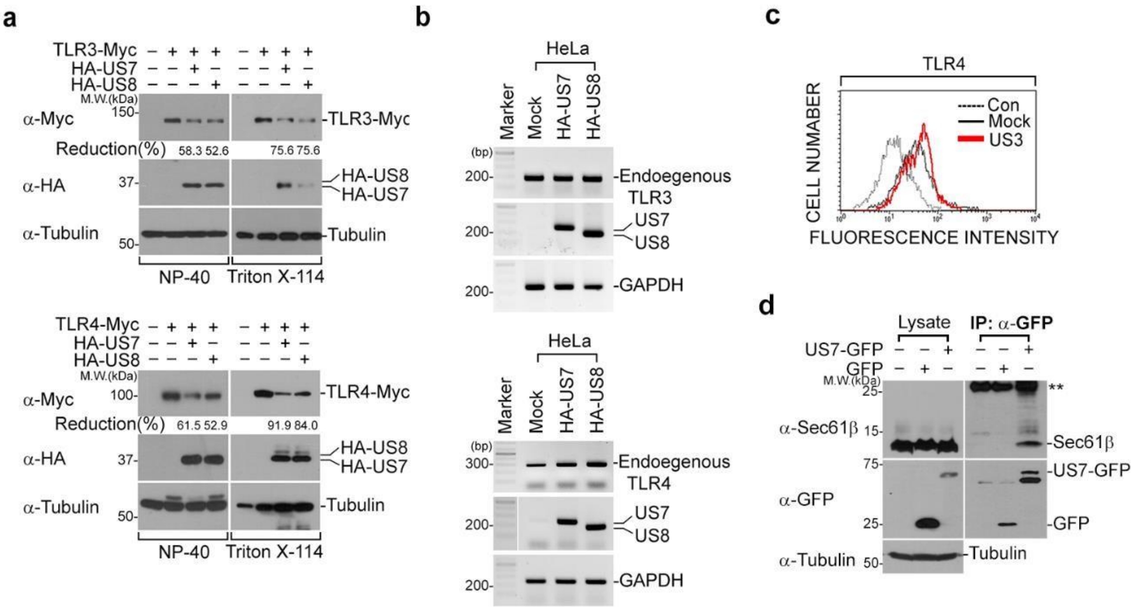

Supplementary Figure 3. US7 and US8 downregulate TLR3 and TLR4 protein expression

(**a**) US7 and US8 degrade TLR3 and TLR4. HeLa cells stably expressing TLR3-Myc or TLR4-Myc were transfected with empty vector, HA-US7 or HA-US8. The lysates were immunoblotted with anti-Myc, anti-HA, or anti-Tubulin antibody. (**b**) The effect of TLR3 or TLR4 mRNA expression in US7- or US8-expressing cells. TLR3 and TLR4 mRNA expression levels from empty vector-, HA-US7-, or HA-US8-expressing HeLa cells were measured by RT-PCR. (**c**) US3 is not required for reducing cell surface expression of TLR4. Endogenous TLR4 surface expression on U937 cells expressing US3 was assessed by FACS analysis. (**d**) Sec61β binds to US7-GFP, but not to GFP alone. Lysates from HEK 293T cells expressing GFP or US7-GFP were immunoprecipitated with anti-GFP antibody prior to immunoblot analysis with indicated antibodies. **Ig light chains. Data are representative of three independent experiments.

Supplementary Figure 4. Park et al.

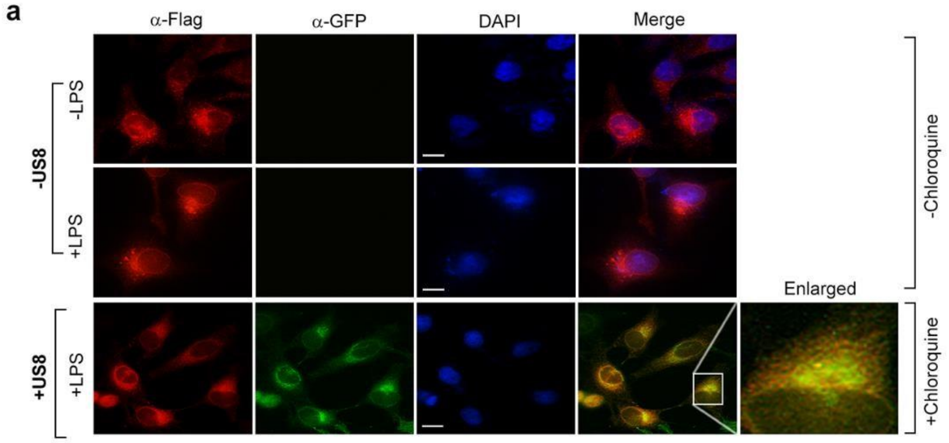

Supplementary Figure 4. US8 colocalizes with TLR4 in lysosomes. HeLa cells expressing Flag-TLR4/MD-2-Myc were transfected with empty vector or US8-GFP and then treated 5 μg ml^{-1} LPS with 100 μM chloroquine for 4 h. Cells were stained with anti-Flag antibody. DAPI was used as a nuclear counterstain. Scale bars, 10 μm. Data are representative of three independent experiments.

Supplementary Figure 5. Park et al.

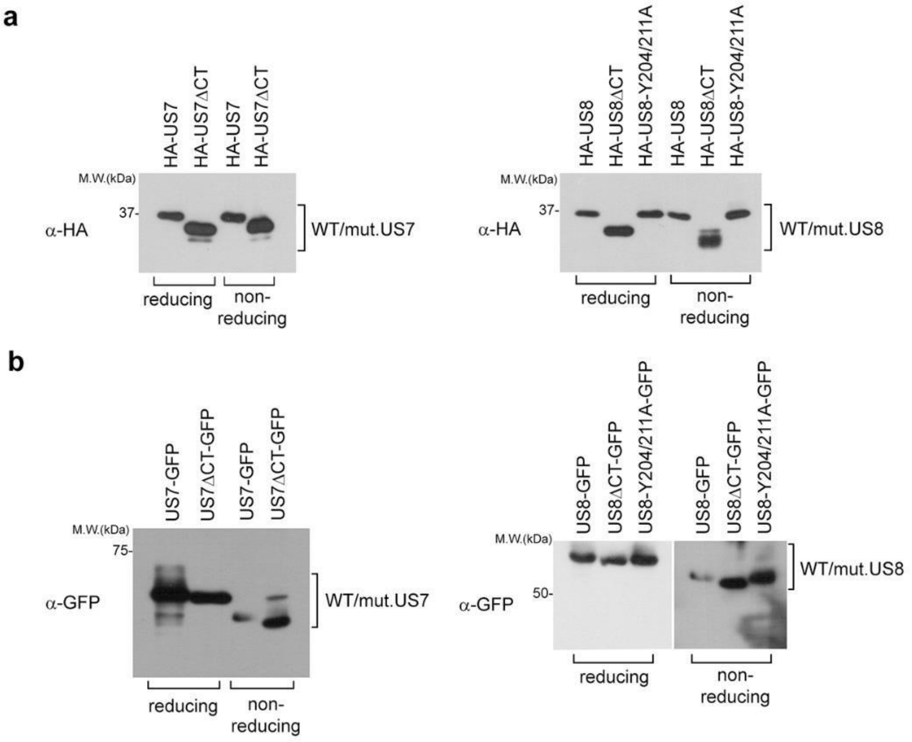

Supplementary Figure 5. Effects of US7 and US8 cytoplasmic domain on folding (a-b) US7GFP-, US7ΔCT-GFP- or US8-GFP-, US8ΔCT-GFP, US8-Y204/211A-GFP-expressing HeLa cells were immunoblotted with anti-HA or anti-GFP antibody under reducing and nonreducing conditions. Data are representative of three independent experiments.

Supplementary Figure 6. Park et al.

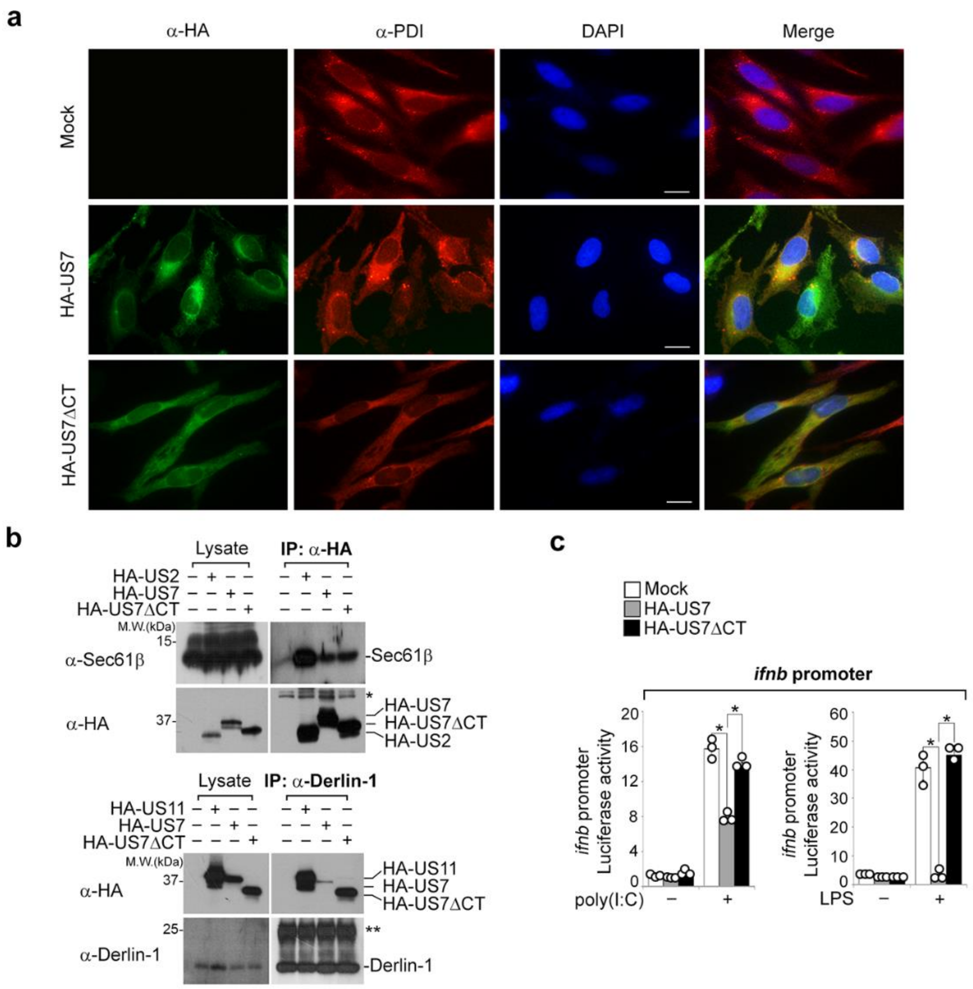

Supplementary Figure 6. Effects of US7 cytoplasmic domain Sec61/Derlin-1 interaction and antiviral response. **(a)** US7 and US7ΔCT localize on the ER. HeLa cells were transfected with empty vector, HA-US7 or HA-US7ΔCT, and then stained with anti-HA and an ER marker (anti-PDI). The nuclei were stained with DAPI. Scale bars, 10 μm. **(b)** The C-terminal region of US7 is dispensable for binding Sec61/Derlin-1. Lysates from HEK 293T cells expressing HA-US7 or HA-US7ΔCT were immunoprecipitated with anti-HA or anti-Derlin-1 antibody prior to immunoblot analysis with indicated antibodies. *Ig heavy chains, **Ig light chains. **(c)** US7ΔCT restores *ifnb* promoter activity. Luciferase assays of *ifnb* promoter activity in HEK 293T expressed empty vector, HA-US7, or US7ΔCT and then incubated with 10 μg ml^{-1} poly(I:C) or 5 μg ml^{-1} LPS for 12 h. *P<0.001 (Student's *t*-test). Data are representative of three independent experiments and are presented as means ± s.d. in **c**.

Supplementary Figure 7. Park et al.

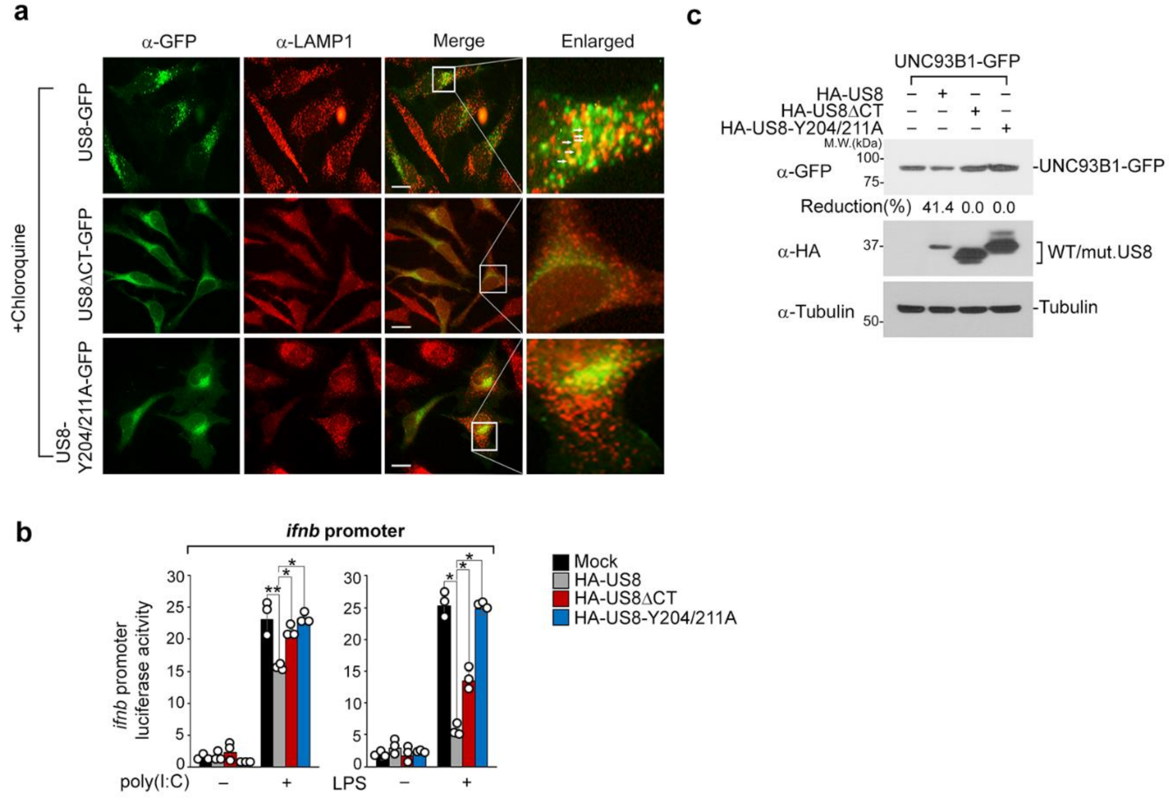

Supplementary Figure 7. Effects of US8 cytoplasmic domain on targeting of itself or its targets to the lysosome. (a) Subcellular localization of wild-type or mutant versions of US8. US8-GFP, US8ΔCT-GFP, or US8-Y204/211A-GFP-expressing HeLa cells were stained with late endosome marker (LAMP1). The nuclei were stained with DAPI. Scale bars, 10 μm. **(b)** Both US8ΔCT and US8-Y204/211A restore *ifnb* promoter activity. Luciferase assays of *ifnb* promoter activity in TLR3- or TLR4/MD2-expressing HEK293T transfected with empty vector, HA-US8, US8ΔCT, or US8-Y204/211A, and then incubated with 10 μg ml^{-1} poly(I:C) or 5 μg ml^{-1} LPS for 12 h. **P*<0.001, ***P*<0.05 (Student's *t*-test). **(c)** US8ΔCT or US8Y204/211A restores UNC93B1 expression levels. UNC93B1-GFP-expressing HeLa cells were transfected with HA-US8, HA-US8ΔCT, or US8-Y204/211A. Lysates were immunoblotted with indicated antibodies. Data are representative of three independent experiments and are presented as means ± s.d. in **b**.

Supplementary Figure 8. Park et al.

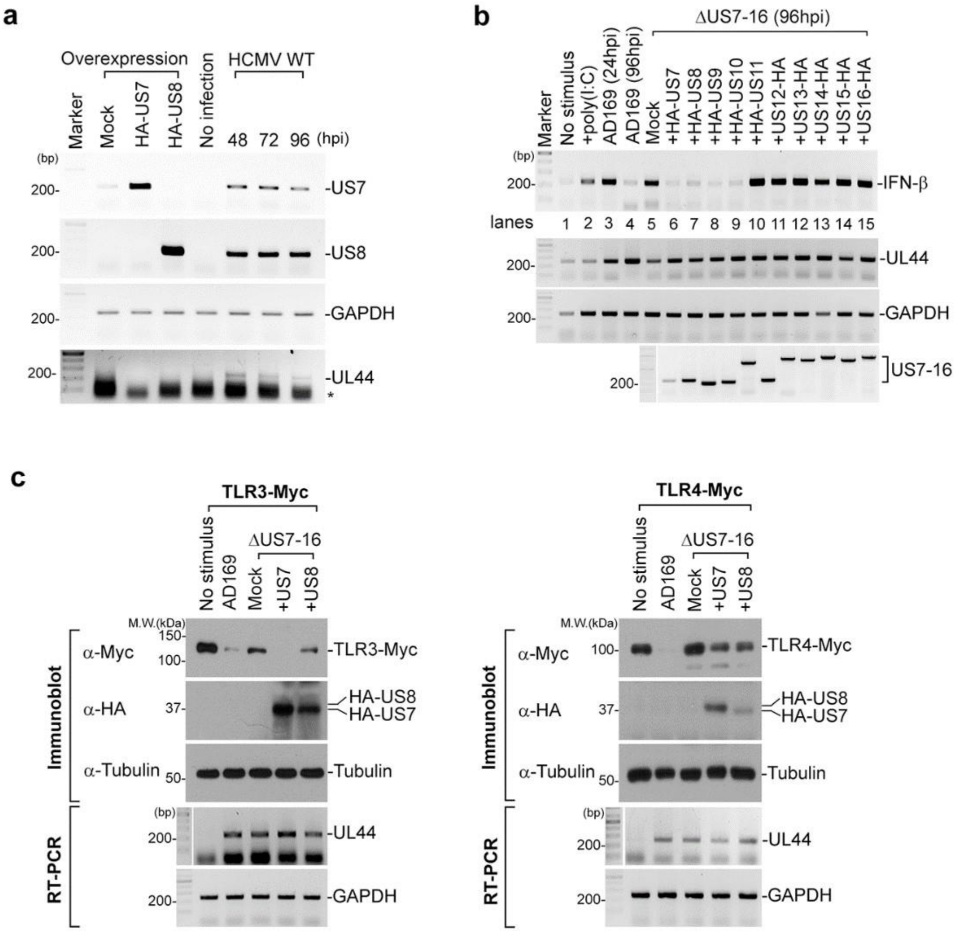

Supplementary Figure 8. HCMV US7 and US8 inhibit antiviral response in vivo (**a**) HFF cells were transfected with HA-US7 or HA-US8 or infected with wild-type HCMV strain. The mRNA expression level of indicated genes were analyzed by RT-PCR. (**b**) US7-US10 proteins attenuate IFN-β expression. HFF cells were infected with HCMV WT HCMVΔUS716 at an MOI of 2 for 96 h and then transfected with the indicated HA-tagged US7-US16 constructs and IFN-β mRNA levels were analyzed by RT-PCR. HFF cells were also treated with 10 μg ml^{-1} poly(I:C) for 24 h were used as a positive control. UL44 and GAPDH were used as HCMV infection and loading controls, respectively. (**c**) HCMVΔUS7-16-Rev.US7 or HCMVΔUS7-16-Rev.US8 degrade TLR3 or TLR4. TLR3-Myc- or TLR4-Myc-expressing HFF cells were infected with HCMV WT or HCMVΔUS7-16 at an MOI of 2 for 96 h and then transfected with HA-US7 or HA-US8. Lysates were immunoblotted with anti-Myc antibody. For a comparison of infection efficacy of wild-type with mutant HCMV, UL44 mRNA levels were measured by RT-PCR. GAPDH was used as a loading control. Data are representative of three independent experiments.

Supplementary Table 1: Sequences of primers

Primers for cloning	Forward Primer Reverse Primer
HA-US7<br>(Xho I / XbaI)	5′- ATT CTC GAG AAA CGT GTG GAA GAC ATG GCG AC -3′<br>5′- ATC TCT AGA TTA GCC CTT GAC AGG ATA GGT CAA AAG -3′
HA-US8<br>(Xho I / XbaI)	5′- CTT GTT AAC ATG AGC AGC AAC GAA TGC TTC AAG -3′<br>5′- ATC GAT CGA TTT AGG CTG TAG CCT CAA TTG TGC A -3′
HA-US7-GFP<br>(Sac I/ BamH I)	5′- ATC GAG CTC GCC ACC ATG GTC CCG TGC A -3′<br>5′- AAA GGA TCC GCC CTT GAC AGG ATA GGT CAA AAG -3′
HA-US8-GFP<br>(Hind III / BamH I)	5′- TGG AAT GAG ACT ATT GTT GAG AA -3′<br>5′- ATT TCC ACT CTG ACT ATG GTC -3′
US7-GFP<br>(Sac I / BamH I)	5′- ATT GAG CTC GCC ACC ATG CGA ATC CAG CTG CTT CTG GTA G -3′<br>5′- A AA GGA TCC GCC CTT GAC AGG ATA GGT CAA AAG -3′
HA-US7ΔCT<br>(BglII/ XbaI)	5′-TGC AAG GAG TGC TGC TAC AAT TGT GGC AA -3′<br>5′- ATT TCT AGA GAC AAT GCA GTA CTG TAG CAG -3′
HA-US3<br>(BglII/ XhoI)	5′- ATT AGA TCT GCC ACC ATG AAG CCG GTG T -3′<br>5- ATT CTC GAG AAT AAA TCG CAG ACG GGC G -3′
HA-US8 ΔCT<br>(BglII/ HpaI)	5′- ATC GAG CTC GCC ACC ATG GTC CCG TGC A -3′<br>5′- ATT GTT AAC TCA CAG CAC GTA TCC CAA CAG CAG -3′
US8 -Y204A	5′- GTG TAT CCA GCG CTT ACG CTC TGC GGT GGC ACG -3′<br>5′- CGT GCC ACC GCA GAG CGT AAG CGC TGG ATA CAC-3′
US8 Y204A/Y211A	5′- GCT GGC CAG GAC TGT GGC CCG TGT ATC CAG CGC TTA -3′<br>5′- TAA GCG CTG GAT ACA CGG GCC ACA GTC CTG GCC AGC -3′
Flag-TLR4-Myc<br>(Hind III/ BamHI)	5′- ATC AAG CTT GCC ACC ATG ATG TCT GCC TCG CGC CTG G -3′<br>5′- ATC GGA TCC GAT AGA TGT TGC TTC CTG CCA ATT GCA T -3′
TLR3-Myc<br>(BglII/ XhoI)	5′- ATT AGA TCT GCC ACC ATG AAG CCG GTG T -3′<br>5′- ATT CTC GAG AAT AAA TCG CAG ACG GGC G -3′
US12-HA<br>(BglII/XhoI)	5′- AAT AGA TCT GCC ACC ATG GTA CAG ATC CAG TTT CAC -3′<br>5′- AAT CTC GAG TTT ATG AAA AAG CCA GTG TGC C -3′
US13-HA<br>(BglII/XhoI)	5′- AAT AGA TCT GCC ACC ATG GAC CCG CCG CTA C -3′<br>5′- AAT CTC GAG CGA GCC ACC GCC ACC T -3′
US14-HA<br>(BglII/XhoI)	5′- ATT AGA TCT GCC ACC ATG GAG ACA GTT TCC ACG C -3′<br>5′- ATT CTC GAG GGC AGC CTT GCT CTG GA -3′
US15-HA<br>(BglII/XhoI)	5′- ATT AGA TCT GCC ACC ATG AGA AGA GAA AAA GGG TTT CA-3′<br>5′-ATT CTC GAG CAG CTT GTC AGA GGA AAA GTA -3′
US16-HA<br>(BglII/XhoI)	5′- ATT AGA TCT GCC ACC ATG GGT CTG CGC TTT CCC-3′<br>5′- ATA CTC GAG GGG CGA GAG GGT GGA C-3′
shSec61β	5′- GAT CCG CAG TAT TGG TTA TGA GTC TTC CGA AGA AGA CTC ATA ACC AAT ACT GTT TTT TGG AAA -3′<br>5′- AGC TTT TCC AAA AAA CAG TAT TGG TTA TGA GTC TTC TTC GGA AGA CTC ATA ACC AAT ACT GCG -3′

shDerlin-1	5′- GAT CCG TGG ATA TGC AGT TGC TGA TCG AAA TCA GCA ACT GCA TAT CCA TTT TTT GGA AA -3′<br>5′- AGC TT TCC AAA AAA TGG ATA TGC AGT TGC TGA TTT CGA TCA GCA ACT GCA TAT CCA CG -3′

UNC93B1-GFP (BglII/EcoRI)	5′- ATT AGA TCT GCC ACC ATG GAG GCG GAG CCG C -3′<br>5′- ATT GAA TTC GAC TGC TCC TCC GGC CC -3′
**RT-PCR primer**	
TLR3	5'- GTA TTG CCT GGT TTG TTA ATT GG -3'<br>5'- GGT ATT GAT CAT GAA AAA GAG TTC -3'
TLR4	5′- CCT ATG AGG ACG ACG ACT ACT ATT AC -3′<br>5′- ATA GAA CGT ACC CTG TAC ACG CCA -3'
IFN-β	5′- CTG ATA ATC CTG ACG AGG GC -3′<br>5′- TGC TCC TTG ATT CTA TGC CG -3′
GAPDH	5'- GAG AAA ATC TGG CAC CAC ACC TTC -3'<br>5'- CGA TTT CCC GCT CGG CCG -3'
UL44	5'- TGA TGA CAT CAA GAA GGT GGT GAA -3'<br>5'- TCC TTG GAG GCC ATG TGG GCC AT -3'
US7	5'- ATC TCA CAC CGT CAG CTG CGT AAT -3'<br>5'- TTC GTA CTG TCC AGA AGA CAA TTG CAC -3'
US8	5′- CCT ATG AGG ACG ACG ACT ACT ATT AC-3′<br>5′- ATA GAA CGT ACC CTG TAC ACG CCA -3′
US9	5'- CGA CTC TCT TAC GTG ATG TTA -3'<br>5'- GAC ACC GAA GCT GAA CAA G -3'
US10	5'- ATG CTA CGC CGG GGA AGC -3'<br>5'- TTC GCG AGG TGG ATA ATA ACC G -3'
US11	5'- ATG AAT CTT GTA ATG CTT ATT CTA-3'<br>5'- CCA CTG GTC CGA AAA CAT C -3'
US12	5′- ATG GTA CAG ATC CAG TTT CAC -3′<br>5′- TTT ATG AAA AAG CCA GTG TGC C -3′
US13	5′- ATG GAC CCG CCG CTA C -3′<br>5′- CGA GCC ACC GCC ACC T -3′
US14	5′- ATG GAG ACA GTT TCC ACG C -3′<br>5′- GGC AGC CTT GCT CTG GA -3′
US15	5′- ATG AGA AGA GAA AAA GGG TTT CA-3′<br>5′- CAG CTT GTC AGA GGA AAA GTA -3′
US16	5′- ATG GGT CTG CGC TTT CCC-3′<br>5′- GGG CGA GAG GGT GGA C-3′
**qPCR primer**	
CXCL10	5'- GAC CTT TCC TTG CTA ACT GC -3'<br>5'- ACT TGA AAT TAT TCC TGC AAG C -3'

TNFSF10	5'- CTA GTG AGA GAA AGA GGT CC -3'<br>5'- ATG AAT GCC CAC TCC TTG AT -3'
IFIT3	5'- TTG GGT GCT GCT ACA AGG-3'<br>5'- AGC ATC AGG GAC TTC CTT AT -3'
CCL8	5'- ACG TGA TCA ATA GGA AAA TTC CT -3'<br>5'- ATA TTT GGT CCA GAT GCT TCA T -3'
ISG15	5'- TGA GGA ATA ACA AGG GCC G -3'<br>5'- ATT CAT GAA CAC GGT GCT CA -3'
CXCL11	5'- CTT GGC TGT GAT ATT GTG TG -3'<br>5'- GTC ACA GTT GTT ACT TGG GTA -3'
