# Supplementary material for: HCMV-encoded US7 and US8 act as antagonists of innate immunity by distinctively targeting TLR-signaling pathways
Source: Nat Commun. 2019 Oct 11;10:4670. doi: 10.1038/s41467-019-12641-4 (PMC6789044; doi:10.1038/s41467-019-12641-4)
Supplement: Supplementary file 4 — Description of Additional Supplementary Files [file 41467_2019_12641_MOESM4_ESM.pdf]

Description of Additional Supplementary Files

File Name: Supplementary Data 1
Description: Raw data showing expression of cellular targets of US7 and US8 in HFF cell expressing US7 or US8 after stimulation by dsDNA.
